# 6-Phosphogluconate dehydrogenase and its crystal structures

**DOI:** 10.1107/S2053230X22001091

**Published:** 2022-02-23

**Authors:** Stefania Hanau, John R. Helliwell

**Affiliations:** a Department of Neuroscience and Rehabilitation, University of Ferrara, Via Borsari 46, Ferrara, Italy; b Department of Chemistry, University of Manchester, Manchester M13 9PL, United Kingdom

**Keywords:** 6-phosphogluconate dehydrogenase, homotropic cooperativity, induced fit, allostery, structure and function, drug targets, bionanotechnology

## Abstract

6-Phosphogluconate dehydrogenase (6PGDH; EC 1.1.1.44) catalyses the oxidative decarboxylation of 6-phosphogluconate to ribulose 5-phosphate in the context of the oxidative part of the pentose phosphate pathway. Depending on the species, it can be a homodimer or a homotetramer. Oligomerization plays a functional role not only because the active site is at the interface between subunits but also due to the interlocking tail-modulating activity, similar to that of isocitrate dehydrogenase and malic enzyme, which catalyse a similar type of reaction. Since the pioneering crystal structure of sheep liver 6PGDH, which allowed motifs common to the β-hydroxyacid dehydrogenase superfamily to be recognized, several other 6PGDH crystal structures have been solved, including those of ternary complexes. These showed that more than one conformation exists, as had been suggested for many years from enzyme studies in solution. It is inferred that an asymmetrical conformation with a rearrangement of one of the two subunits underlies the homotropic cooperativity. There has been particular interest in the presence or absence of sulfate during crystallization. This might be related to the fact that this ion, which is a competitive inhibitor that binds in the active site, can induce the same 6PGDH configuration as in the complexes with physiological ligands. Mutagenesis, inhibitors, kinetic and binding studies, post-translational modifications and research on the enzyme in cancer cells have been complementary to the crystallographic studies. Computational modelling and new structural studies will probably help to refine the understanding of the functioning of this enzyme, which represents a promising therapeutic target in immunity, cancer and infective diseases. 6PGDH also has applied-science potential as a biosensor or a biobattery. To this end, the enzyme has been efficiently immobilized on specific polymers and nanoparticles. This review spans the 6PGDH literature and all of the 6PGDH crystal structure data files held by the Protein Data Bank.

## Abstract 2

6-Phosphogluconate dehydrogenase is in a process of a continuous discovery: despite the determination of several crystal structures over thirty years, a clear elucidation of its mechanism from the structure–function point of view has not yet been reached.

## 1. Introduction

### 1.1. The metabolic role of 6-phosphogluconate dehydrogenase

**Figure 1:**
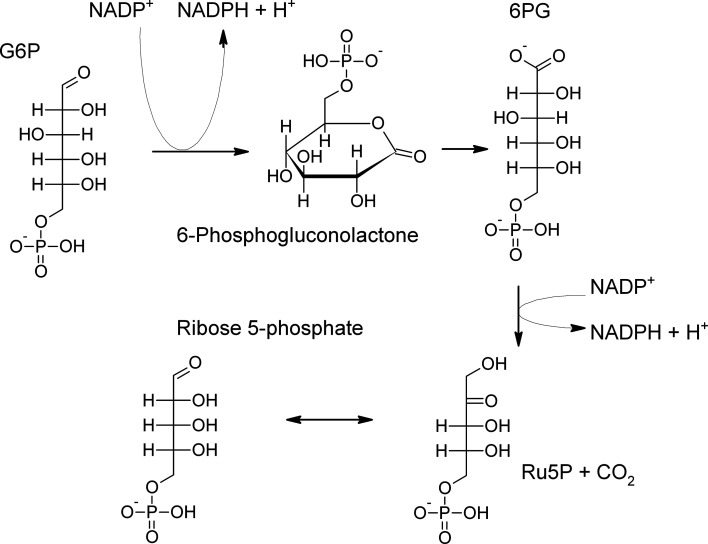
Oxidative part of the pentose phosphate pathway and isomerization between ribulose 5-phosphate (Ru5P) and ribose 5-phosphate. G6P, glucose 6-­phosphate; 6PG, 6-phosphogluconate.

6-Phosphogluconate dehydrogenase (EC 1.1.1.44) catalyses the third reaction in the oxidative part of the pentose phosphate pathway (PPP): the oxidative decarboxylation of 6-phosphogluconate (6PG) to ribulose 5-phosphate (Ru5P) (Fig. 1 ▸). Various abbreviations have been used for this enzyme, such as 6PGDH, 6PDH, PGDH, PDH and 6PGD; while *gnd* is the acronym of the gene, here the abbreviation 6PGDH is employed.

As part of this branch of the PPP, 6PGDH is important for the production of NADPH, which is necessary for reductive biosynthesis, such as the formation of lipids and nucleotides, and the activity of enzymes involved in maintaining cell integrity, in combatting oxidative stress and in the first line of immunological defence. These enzymes include glutathione (GSH) reductase, GSH peroxidase and the NADPH oxidase family (Rada & Leto, 2008 ▸; Li *et al.*, 2019 ▸). NADPH is necessary for redox signalling by, for instance, thioredoxins, glutaredoxins and peroxiredoxins, regulating signal transduction and protein functions implicated in proliferation, apoptosis, inflammation and mitochondrial integrity (Hanschmann *et al.*, 2013 ▸). Also, ribose 5-phosphate (R5P) produced by R5P isomerase in the non-oxidative part of the PPP is important in nucleotide synthesis and together with Ru5P participates in the reversible interconversion of metabolites, producing xylulose 5-phosphate, sedoheptulose 7-phosphate, erythrose 4-phosphate and the glycolytic/gluconeogenetic intermediates glyceraldehyde 3-phosphate and fructose 6-phosphate. Enzymes in the non-oxidative PPP include, among others, transketolase and transaldolase (Kabashima *et al.*, 2003 ▸; Stincone *et al.*, 2015 ▸; Bommer *et al.*, 2020 ▸).

Classically, the main regulatory enzyme of the PPP is considered to be the first dehydrogenase, namely glucose 6-phosphate dehydrogenase (G6PDH), the activity of which is basically inhibited by NADPH (Christodoulou *et al.*, 2019 ▸). Nevertheless, the power of 6PG to inhibit the glycolytic enzyme phosphoglucose isomerase (Parr, 1956 ▸) shows it to be a key metabolic regulator, increasing carbon flux through the PPP with respect to glycolysis when required (Dubreuil *et al.*, 2020 ▸). The interesting role of 6PG as a modulator of CD8^+^ T-cell activation and differentiation, as shown by either pharmacological inhibition or genetic *gnd* ablation, indicated 6PGDH to be a promising therapeutic target to strengthen immunity (Daneshmandi *et al.*, 2021 ▸). Also, in another study ablation of *gnd* revealed the activating effect of Ru5P on lipogenesis by means of final inhibition of the specific phosphorylation of acetyl-CoA carboxylase 1 by adenine monophosphate-activated protein kinase (AMPK). Ru5P has been shown to directly inhibit the formation of the AMPK-activating complex between tumour suppressor liver kinase B1 (LKB1), pseudokinase Ste20-related adaptor (STRAD) and scaffolding-like adaptor mouse protein 25 (MO25) (Lin *et al.*, 2015 ▸).

Given its importance in metabolism, 6PGDH has been studied as a drug target in cancer (Hitosugi *et al.*, 2012 ▸; Lin *et al.*, 2015 ▸; Yang *et al.*, 2018 ▸) and in a number of infectious diseases (Barrett & Gilbert, 2002 ▸; Hanau *et al.*, 2004 ▸, 2007 ▸; Esteve & Cazzulo, 2004 ▸; González *et al.*, 2011 ▸; Kerkhoven *et al.*, 2013 ▸; Haeussler *et al.*, 2018 ▸; Wang *et al.*, 2021 ▸; Jakkula *et al.*, 2021 ▸), attempting to exploit differences between the host enzyme and the homologous microbial enzyme (Hanau *et al.*, 1996 ▸; Bertelli *et al.*, 2001 ▸; Dardonville *et al.*, 2003 ▸, 2004 ▸; Montin *et al.*, 2007 ▸; Ruda *et al.*, 2010 ▸; Morales-Luna *et al.*, 2021 ▸). Also, 6PGDH is a target for enzyme and metabolic engineering, with the aim of improving the productivity of biocatalysts for useful compounds, such as l-lysine and riboflavin (Ohnishi *et al.*, 2005 ▸; Wang *et al.*, 2011 ▸).

### 1.2. Different types of 6PGDH

Some bacterial 6PGDHs are specific for NAD^+^, while others can use both NAD^+^ and NADP^+^ (Maturana *et al.*, 2021 ▸). In the thermostable 6PGDH from the hyperthermophilic bacterium *Thermotoga maritima* the coenzyme preference could be reversed from NADP^+^ to NAD^+^ by site-directed mutagenesis for applications in biobatteries (Chen *et al.*, 2016 ▸). Another biotechnological application is the use of 6PGDH as a biosensor (Cetó *et al.*, 2011 ▸), and for this reason the enzyme has been efficiently immobilized on specific polymers and nanoparticles (Albanese *et al.*, 2014 ▸; Sahin, 2019 ▸). 6PGDH is mainly cytosolic, although isoforms are present in organelles such as peroxisomes and chloroplasts (Krepinsky *et al.*, 2001 ▸; Strijbis *et al.*, 2012 ▸). Furthermore, membrane-associated 6PGDHs have been reported in some bacteria (Daniely *et al.*, 2006 ▸; Sarmiento-Pavía *et al.*, 2021 ▸). It is now recognized that there are both homodimeric and homotetrameric 6PGDHs. The latter group are mainly found in prokaryotes, although a dimer–tetramer equilibrium affected by ligands has been reported for 6PGDH from the protist *Trypanosoma brucei* (Tsai & Chen, 1998 ▸; Hanau *et al.*, 2013 ▸; Maturana *et al.*, 2021 ▸; Sarmiento-Pavía *et al.*, 2021 ▸).

## 2. Catalytic mechanism

**Figure 2:**
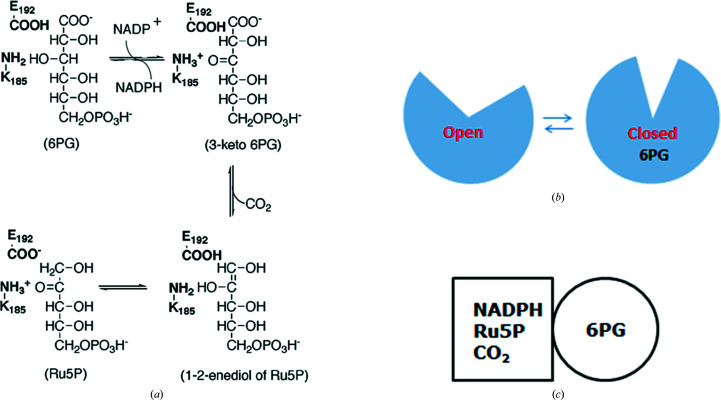
(*a*) 6PGDH-catalysed reaction and the two main amino-acid residues involved (residue numbers in *T. brucei* 6PGDH). (*b*) Scheme of the isomerization step between the open and closed conformation of 6PGDH. (*c*) Scheme of the asymmetric conformation of the protein, as shown by affinity-labelling and kinetic studies, in which the two different subunit conformations are represented as different shapes.

6PGDH does not require a divalent metal ion for activity, and an acid–base mechanism was indicated by the pH dependence of the kinetic parameters and the dissociation constants of competitive inhibitors (Berdis & Cook, 1993*b* ▸; Hanau *et al.*, 1996 ▸; Price & Cook, 1996 ▸), while covalent catalysis by the formation of a Schiff-base intermediate has been ruled out (Topham & Dalziel, 1986 ▸). Kinetic studies showed a sequential reaction mechanism with the formation of a ternary complex, and random order for both substrate and product binding, in several 6PGDHs (Berdis & Cook, 1993*a* ▸; Hanau *et al.*, 1996 ▸; Price & Cook, 1996 ▸; Wang *et al.*, 2002 ▸). The reaction proceeds by three main catalytic steps: first oxidation, then decarboxyl­ation of the 3-keto-6PG intermediate as the second step, and finally tautomerization of the enediolic form of Ru5P to the ketose form as the third step (Fig. 2 ▸ *a*). This stepwise mechanism was elucidated by different methodological approaches such as the use of the substrate analogue 2-deoxy-6PG, tritium tracking and multiple isotope effects (Lienhard & Rose, 1964 ▸; Rippa *et al.*, 1972 ▸; Rendina *et al.*, 1984 ▸; Hanau *et al.*, 1992*a* ▸). There is not only one rate-limiting step; in fact, a step preceding hydride transfer, hydride transfer itself, decarboxylation of the keto intermediate and a step after enol–keto tautomerization of Ru5P also contribute to rate limitation. This statement is based on analysis of deuterium, tritium and ^13^C isotope effects in sheep liver and *Candida utilis* 6PGDHs (Hwang *et al.*, 1998 ▸; Hwang & Cook, 1998 ▸; Hanau *et al.*, 2010 ▸).

As for a great many enzymes, 6PGDH was studied long before the first crystal structures were obtained. Chemical modification, above all of yeast 6PGDH, showed that, among others, a lysine and a histidine residue were present in the active site (Rippa *et al.*, 1967 ▸; Rippa & Pontremoli, 1968 ▸). Also, substrate binding causes increased stability towards proteolysis, denaturing agents and chemical modification; in particular, the reactivity of all cysteines is cancelled (Rippa *et al.*, 1978 ▸; Hanau *et al.*, 2014 ▸). This was a strong indication of an isomerization step between open and closed conformations of 6PGDH, with the latter involved in catalysis of the redox step. Mutagenesis and isotopic effects corroborated these findings (Fig. 2 ▸ *b*; Cervellati *et al.*, 2005 ▸, 2008 ▸; Li & Cook, 2006 ▸).

### 2.1. Asymmetry in structure–function studies

In 6PGDH from different sources, only one NADP^+^ molecule is bound in the enzyme dimer during the formation of ternary complexes with either the substrate or intermediate analogues. A special case is erythrocytic 6PGDH, which shows coenzyme half-sites reactivity with NADPH even in the absence of 6PG (Rippa *et al.*, 1979 ▸, 1998 ▸, 2000 ▸; Dallocchio *et al.*, 1985 ▸; Montin *et al.*, 2007 ▸). The first evidence of co­enzyme half-sites reactivity came from affinity-labelling studies with a dialdehydic NADP^+^ analogue that inactivates 6PGDH. It was found that this was due to the covalent binding of two moles of inhibitor per mole of dimer, while only one mole was bound in the presence of 6PG (Rippa *et al.*, 1975 ▸; Hanau *et al.*, 1992*b* ▸). Furthermore, stopped-flow studies of the first turnover of sheep liver 6PGDH showed the production of only one NADPH molecule per dimer (Topham *et al.*, 1986 ▸). Confirmation of this asymmetric behaviour also came from the negative cooperativity for NADP^+^ found in 6PGDH from human erythrocytes and rat liver (Dallocchio *et al.*, 1985 ▸; Voinova *et al.*, 1996 ▸).

Studies of the partial reaction of decarboxylation of 3-keto-2-deoxy-6PG and the reverse reaction, the reductive carboxylation of Ru5P, using kinetic isotope effects showed homotropic allosteric modulation by the substrate (Fig. 2 ▸ *c*). This improves the catalytic efficiency, and might originate in fine-tuning of the 6PGDH activity favouring metabolic co­ordination between the PPP and the glycolytic pathway (Hanau *et al.*, 1992*a* ▸, 1993*a* ▸, 2010 ▸). Several potential regulatory mechanisms have been revealed: 6PG not only inhibits phosphoglucose isomerase in some species but also activates phosphofructokinase (Parr, 1956 ▸; Sommercorn & Freedland, 1982 ▸). Besides, although fructose-1,6-bisphosphate (F1,6BP) and 3-phosphoglycerate inhibit 6PGDH, in neural cells it has been shown that F1,6BP causes an increased flux of glucose into the PPP (Dyson & D’Orazio, 1973 ▸; Kelleher *et al.*, 1995 ▸; Hitosugi *et al.*, 2012 ▸).

**Figure 3:**
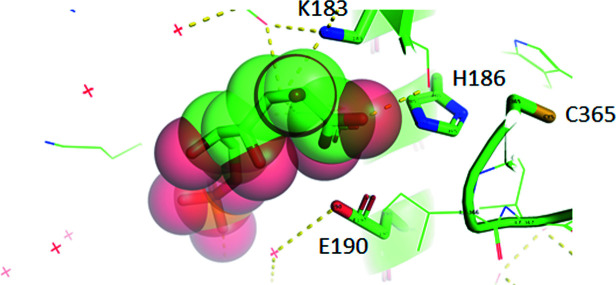
Selection of residues within 8 Å of 6PG (shown as both sticks and van der Waals spheres) in the sheep 6PGDH–6PG complex (PDB entry 1pgp) implied in changes of ionization upon substrate binding (created using *PyMOL*).

Regarding the structure–function relationship of 6PGDH, not only the coenzyme half-sites reactivity but also the homotropic allostery of 6PG indicates the presence of an asymmetric conformation of the protein. This would allow either the decarboxylation step or the binding of Ru5P in one subunit, while the other subunit binds 6PG as an effector. In fact, there is only one substrate-binding site per subunit, which is at an interface made up of residues from both subunits, thus easily allowing inter-subunit communication (Fig. 3 ▸ *b*; Adams *et al.*, 1994 ▸; Rippa *et al.*, 1998 ▸, 2000 ▸; Hanau *et al.*, 1993*a* ▸, 2010 ▸).

### 2.2. Catalytic residues

Coming back to the accepted chemical mechanism of catalysis by 6PGDH (Fig. 2 ▸ *a*), an enzyme general base in the first step accepts a proton from the 3-hydroxyl group of 6PG, concomitant with hydride transfer to NADP^+^. In the second step, the same general base shuttles the proton between itself and the C3 carbonyl group to allow decarboxylation of the 3-keto-6PG intermediate, which generates the enediol form of Ru5P. In the third step, an enzyme general acid donates a proton to C-1 of the enediol and the general base again abstracts a proton from C-2 to catalyse tautomerization, giving the ketose Ru5P (Berdis & Cook, 1993*a* ▸; Wang & Li, 2006 ▸). Thus, the protonation states of the catalytic base and acid are inverted at the end of the reaction compared with those in the enzyme upon substrate binding (Fig. 2 ▸ *a*). This is confirmed by the perturbation of p*K*_a_ obtained in the log *V* curves compared with the log *V*/*K*_NADP_ profiles indicating p*K*_a_ values for ion­ization of groups in the enzyme–6PG complex. This finding was corroborated by measurement of the hydrogen ions released in calorimetric binding studies of wild-type *T. brucei* 6PGDH and mutants of the catalytic residues (Hanau *et al.*, 1996 ▸, 2014 ▸; Price & Cook, 1996 ▸; Montin *et al.*, 2007 ▸). The proton balance not only confirms the half-sites mechanism of 6PGDH but also shows the dissociation of other groups upon substrate binding, which are most probably involved in the open to closed conformation change of the enzyme subunits (Hanau *et al.*, 2014 ▸). The first solved 6PGDH crystal structure, from sheep liver, demonstrated that the conserved Lys183 (185 in *T. brucei* 6PGDH) is positioned to act as the general base (Figs. 3 ▸ and 4 ▸) and site-directed mutagenesis confirmed its role (Adams *et al.*, 1994 ▸; Zhang *et al.*, 1999 ▸; Hanau *et al.*, 2014 ▸). The role of Glu190 (Figs. 3 ▸ and 4 ▸; 192 in *T. brucei* 6PGDH) as the general acid has mainly been shown by site-directed mutagenesis (Karsten *et al.*, 1998 ▸; Hanau *et al.*, 2014 ▸). Moreover, both Lys185 and Glu192 mutants of *T. brucei* 6PGDH no longer present coenzyme half-sites reactivity during formation of the ternary complex. In fact, the *T. brucei* enzyme binds two NADP^+^ molecules per dimer, while in the ternary complex with substrate and intermediate analogues [5-phospho-d-ribonate and 4-phospho-d-erythronate (4PE), respectively] only one coenzyme molecule binds per dimer (Montin *et al.*, 2007 ▸). Conversely, Lys185His, Lys185Arg and Glu192Gln mutants do not display a significant decrease in the binding stoichiometry of the oxidized coenzyme in the presence of 4PE (unpublished data).

**Figure 4:**
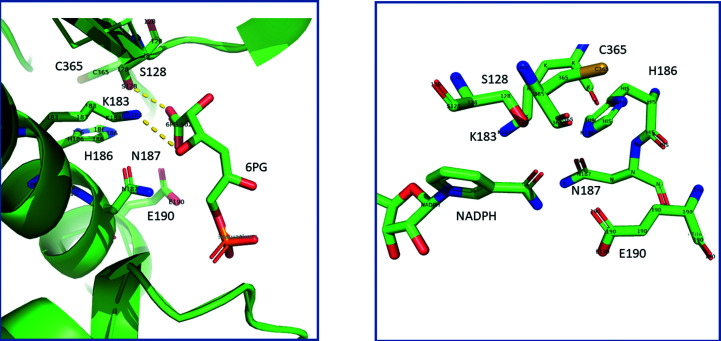
Close-up view of the active site highlighting the His186, Ser128 and Asn187 triad in the sheep liver 6PGDH–6PG complex (left; some polar contacts are shown as dashed lines; PDB entry 1pgp) and in the enzyme–NADPH complex (right; PDB entry 1pgo) (created using *PyMOL*).

His186 and Cys365 (as numbered in the sheep sequence) are conserved residues that are positioned within 8 Å of the active site (Fig. 3 ▸). A comparison of wild-type *T. brucei* 6PGDH and its mutants at the corresponding His188 and Cys372 showed a change in proton release and in cysteine reactivity upon 6PG binding, as well as a decrease in activity, a p*K*_a_ perturbation and no half-sites reactivity (unpublished data). Furthermore, mutagenesis of Cys365 in sheep 6PGDH demonstrates that it is the fast-reacting cysteine, which is thus unprotonated in the apoenzyme while changing ionization during the reaction pathway (Cervellati *et al.*, unpublished data; Hanau *et al.*, 1992*b* ▸). These findings suggest that these residues are involved in the 6PG-induced ionization changes that are required for catalysis and are implied in switching to the correct conformation. Cysteine nitrosylation of *Plas­modium falciparum* 6PGDH has been shown to decrease enzyme activity (Haeussler *et al.*, 2018 ▸). Moreover, His185 in *Staphylo­coccus aureus* 6PGDH (corresponding to His186 in the sheep enzyme) is the target of enzyme inactivation by Ag^+^ (PDB entry 7cb6); this residue, Ser128 and Asn187 have been proposed as a triad controlling the equilibrium between the open and closed forms based on mutagenesis and isotopic effects (Fig. 4 ▸; Li *et al.*, 2006 ▸; Wang *et al.*, 2021 ▸).

## 3. Crystallographic structures

In Table 1 ▸, the cited structures, PDB codes and details are reported. This includes a column in which peaks unmodelled in the PDB entry have been inspected in the *Coot* molecular-graphics visualization system (Emsley *et al.*, 2010 ▸). It also includes a column in which the PDB validation reports are assessed by clashscore. Specific comments of interest based on the PDB report are also provided in these columns.

### 3.1. The first structure of 6PGDH solved by X-ray crystallography

**Figure 5:**
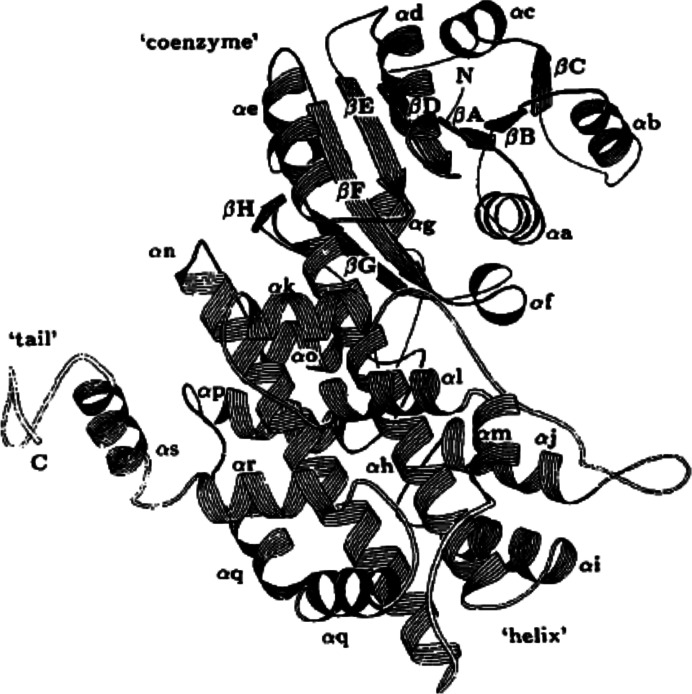
Historical ribbon diagram of ovine 6PGDH monomer (PDB entry 2pgd; reproduced from Adams *et al.*, 1991 ▸).

**Figure 6:**
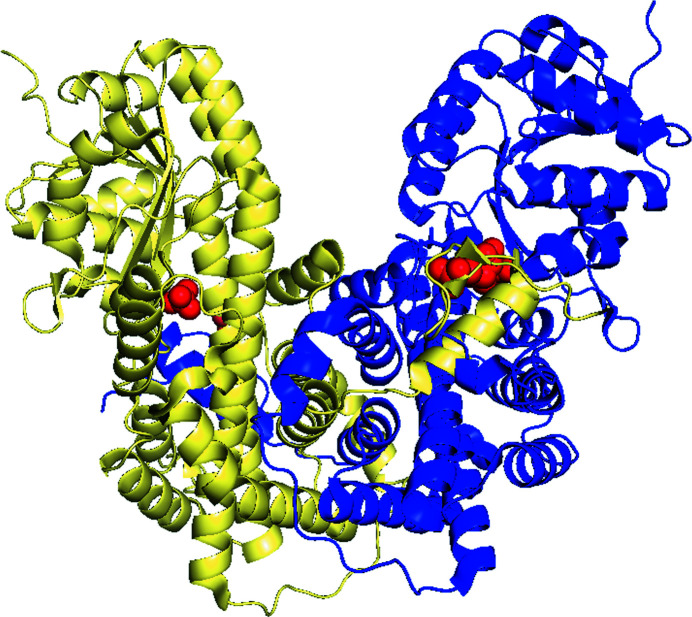
Ribbon diagram of a *G. stearothermophilus* 6PGDH homodimer (PDB entry 2w90) with 6PG and two sulfates, shown as red spheres, bound in the blue and green subunits, respectively (created using *PyMOL*).

The first structure of 6PGDH to be solved by X-ray crystallo­graphy was that from *Ovis aries* (PDB entry 2pgd) in the laboratory of M. J. Adams. The studies proceeded as per the methods of the day at resolutions of 6 Å in 1977 followed by 2.6 Å in 1983, and finally 2.5 and 2 Å, based on a revised sequence, in 1991 and 1995, respectively. It was a homodimer with twofold symmetry, so that a rotation by 180° reproduces exact copies of the molecules (Figs. 5 ▸ and 6 ▸, Table 1 ▸). Each subunit is formed by three domains. The coenzyme-binding amino-terminal domain contains a typical dinucleotide Rossmann β–α–β fold followed by a short helix and an additional β–α–β unit antiparallel to this fold. The central helical domain consists of two large antiparallel helices packed against each other and enclosed on either side by four small helices (Figs. 5 ▸ and 7 ▸). Finally, the carboxy-terminal tail penetrates the other subunit (Fig. 6 ▸) (Adams *et al.*, 1977 ▸, 1978 ▸, 1983 ▸, 1991 ▸, 1994 ▸; Abdallah *et al.*, 1979 ▸; Phillips *et al.*, 1995 ▸).

The solved complex with 6PG (PDB entry 1pgp) showed that the substrate-binding site was made up of residues from both subunits. The carboxyl group of 6PG makes hydrogen bonds to Ser128 in the βF–αf loop of the coenzyme domain and to Glu190 in the large helical domain, and the 6-phosphate binds to Arg446 in the αs in the tail of the second subunit. In the apoenzyme crystal a tightly bound sulfate is in the same position as the 6-phosphate of 6PG would be and another less tightly bound sulfate that is at the border with the coenzyme domain is also displaced by 6PG (Adams *et al.*, 1994 ▸). Globally, four amino acids of the substrate neighbours come from the coenzyme domain, ten from the helical domain and two from the tail of the other subunit (Phillips *et al.*, 1998 ▸). The importance of the tail was demonstrated for *Saccharomyces cerevisiae* 6PGDH, with truncated mutants lacking 35, 39 or 53 C-terminal residues losing activity despite remaining as homodimers (PDB entry 2p4q; He *et al.*, 2007 ▸).

**Figure 7:**
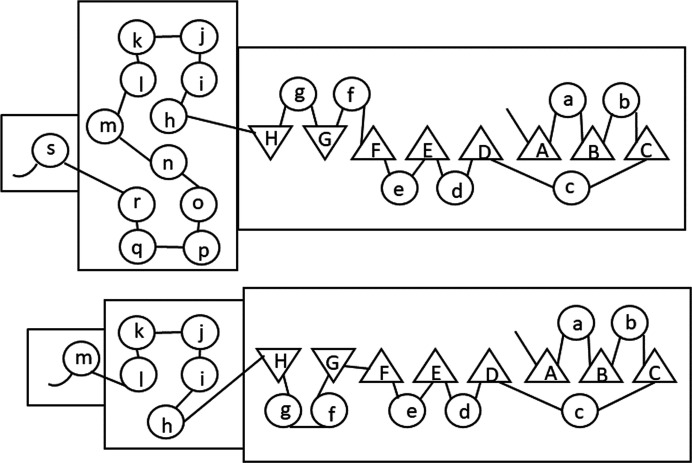
Topology diagram with sheet strands (triangles) and helices (circles) of sheep liver 6PGDH (top) and a common topology diagram of short-chain 6PGDHs and HIBADH (bottom) (Adams *et al.*, 1991 ▸; Sarmiento-Pavía *et al.*, 2021 ▸).

The central helical domain with 11 helices represented a new motif with a duplicated five-helix segment (Fig. 7 ▸) and with only the first copy of the motif providing active-site residues, while both are involved in the dimer interface (Phillips *et al.*, 1995 ▸). The central part with αm joins the repeats by forming an αm–αn external loop (see Figs. 5 ▸ and 7 ▸ for numbering/labelling; Phillips *et al.*, 1998 ▸).

#### 3.1.1. The N-terminal domain

The dinucleotide coenzyme-binding fingerprint sequence in the βa–αa turn was Gly-*X*-Ala-*X*-Met-Gly (residues 9–14), while it was previously found to be Gly-*X*-Gly-*X*-*X*-Gly in NAD^+^-binding dehydrogenases and Gly-*X*-Gly-*X*-*X*-Ala in several NADP^+^-specific dehydro­genases. This showed that only the first glycine is actually needed to form the tight turn, interacting with the pyrophos­phate (Adams *et al.*, 1991 ▸; Lokanath *et al.*, 2005 ▸).

**Figure 8:**
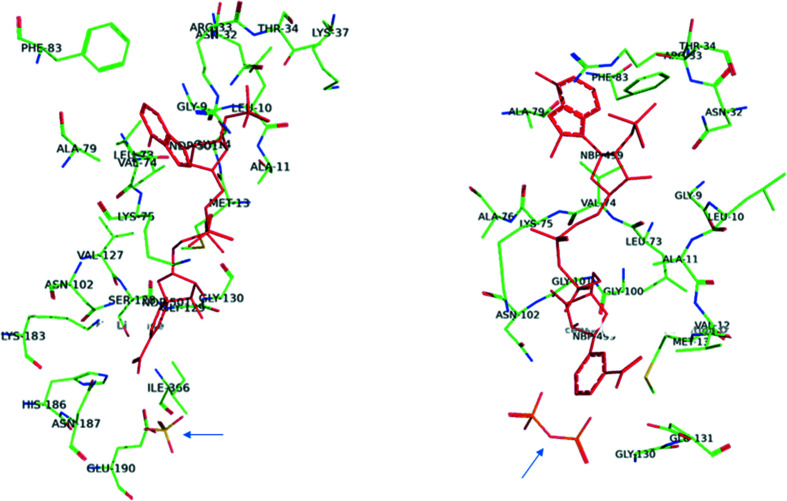
Binding modes of NADPH (left; PDB entry 1pgo) and nicotinamide-8-bromoadenine dinucleotide phosphate (Nbr^8^ADP, an analogue of NADP^+^; right; PDB entry 1pgn) to ovine 6PGDH. One sulfate is present in the substrate-binding site on the left while a pyrophosphate is on the right, as indicated by arrows (created using *PyMOL*).

Conversely, the cornerstone structures of the binary complexes with NADPH (PDB entry 1pgo), nicotinamide-8-bromoadenine dinucleotide phosphate (Nbr^8^ADP; PDB entry 1pgn) and 2′AMP (PDB entry 1pgq) highlighted the role of Asn32, Arg33 and Thr34 in the turn between βb and αb in interacting with the 2′-phosphate mainly by hydrogen bonds and defining NADP^+^ specificity (Fig. 8 ▸). Upon binding the coenzyme, the side chain of Arg33 orders, forming one side of the adenine-binding pocket. The other side is defined by hydrophobic residues in αd and in the loop between βd and αd (Figs. 5 ▸ and 8 ▸; Adams *et al.*, 1994 ▸). Mutagenesis of the conserved arginine and asparagine in *Lactococcus lactis* and *Gluconobacter oxydans* 6PGDH demonstrated their role in specificity for NADP^+^ over NAD^+^, while showing that aspartate, which is present in NAD^+^-dehydrogenases in place of the asparagine, hinders placement of the 2′-phosphate (Tetaud *et al.*, 1999 ▸; Maturana *et al.*, 2021 ▸). Among the residues in the coenzyme-binding site, Lys76 (Lys75 in the sheep structure; Fig. 8 ▸) has been shown to be acetylated by dihydrolipoamide *S*-acetyltransferase in cancer cells, which causes an increase in NADP^+^ affinity and upregulation of 6PGDH (Shan *et al.*, 2014 ▸). Furthermore, these structures showed that the nico­tinamide ring rotates around the *N*-glycosidic bond after reduction of the coenzyme (from the *syn* to the *anti* conformation) and bound NADPH is more extended than the NADP^+^ analogue (Fig. 8 ▸). Mutagenesis confirmed that Met13 and Glu131 are necessary to orientate NADP^+^ such that a hydride can be transferred from the C3 of 6PG, while in the crystals of the NADPH complex distinct residues are in contact with the nicotinamide ribose (Asn102) and the nicotinamide (Ser128, Gly129, Gly130, His186, Asn187, Lys183 and Glu190) (Figs. 4 ▸ and 8 ▸; Adams *et al.*, 1994 ▸; Cervellati *et al.*, 2005 ▸). Since these residues are also involved in the binding of 6PG, movements of several of these amino acids should occur during dehydrogenation, which is judged to be consistent with a shift from the closed to the open conformation of the enzyme. Accordingly, it has been suggested that the nicotin­amide flip, which causes a change in the position of the proton donor Glu190, which was previously hydrogen-bonded to the 6PG carboxylate (Figs. 4 ▸ and 8 ▸), might facilitate decarboxyl­ation and tautomerization. This has in fact been shown to be activated by nonreducing NADPH analogues (Hanau *et al.*, 1992*a* ▸; Rippa *et al.*, 2000 ▸; Cervellati *et al.*, 2005 ▸).

A glycine-rich tight turn between βh and αh, with Asp176 in the middle being the only residue in a disallowed region of the Ramachandran plot for the apoenzyme with three sulfates bound, forms the junction between the coenzyme and the helix domain. Thus, it has been shown to be in a good position to act as a hinge in any functional domain-closure event (Phillips *et al.*, 1995 ▸). In tetrameric *Thermus thermophilus* 3-hydroxyisobutyrate dehydrogenase (HIBADH; PDB entry 2cvz), which belongs to the same superfamily as 6PGDH, Lokanath and coworkers reported an induced-fit interdomain rearrangement, with a 12° orientational difference between the open and NADP^+^-bound closed conformations (Lokanath *et al.*, 2005 ▸).

#### 3.1.2. The active site

The crystal structure of 6PGDH complexed with 6PG (PDB entry 1pgp) revealed the residues binding the substrate, which were subsequently found to be highly conserved in the sequences of other 6PGDHs (Tetaud *et al.*, 1999 ▸; Igoillo Esteve & Cazzulo, 2004 ▸; Cameron *et al.*, 2009 ▸; González *et al.*, 2011 ▸; Jakkula *et al.*, 2021 ▸). Apart from Ser128 and Glu190, which make hydrogen bonds to the carboxyl group, Gly129 and Gly130 are in the region of the O atoms bound to C1 and C2 of 6PG. Moreover, apart from the catalytic Lys183 and the nearby conserved His186 and Asn187 (Figs. 3 ▸ and 4 ▸), Tyr191, Lys260 and Arg287 are in the region of the 6-phosphate of 6PG, while Arg446 and His452 come from the other subunit. Some key residues such as Ser128, Gly129 and Gly130, Lys183 and Asn187 have also been recognized to be highly conserved in dehydrogenases such as HIBADH (Ser124, Gly124 and Gly125, Lys173 and Asn177 in the rat sequence), which revealed that the β-hydroxyacid dehydro­genase (β-HADH) superfamily, including the HIBADH group and the PGDH group, has no requirement for divalent metal ions (Hawes *et al.*, 1996 ▸; Lokanath *et al.*, 2005 ▸; Park *et al.*, 2016 ▸). The lysine and asparagine are inside the so-called catalytic motif identified in the superfamily structural tree (Maturana *et al.*, 2021 ▸). Specific patterns of the superfamily are the presence of both an N-terminal α/β domain with an additional extension compared with the coenzyme-binding Rossmann fold of other oxidoreductase families and an all-α domain. The finding in recent years of two main subfamilies in the 6PGDH group, the long-chain and short-chain 6PGDHs (with subunit average weights of about 52 and 35 kDa, respectively), with only prokaryotic species presenting short-chain 6PGDHs, suggest that the phylogenetic origin of 6PGDH is the β-HADH gene. In fact, both HIBADH and short-chain 6PGDHs do not present duplication in the all-helix domain (Fig. 7 ▸). Thus, it is most probable that 6PGDH evolved from β-HADH, following gene-duplication and domain-deletion events in long-chain 6PGDHs (Andreeva & Murzin, 2006 ▸; Pickl & Schönheit, 2015 ▸; Sarmiento-Pavía *et al.*, 2021 ▸; Maturana *et al.*, 2021 ▸).

#### 3.1.3. The tail and the dimer interface

Residues of the small tail, which is threaded in the loop between αm and αn of the other subunit, contribute to the substrate-binding and coenzyme-binding pockets. This loop has been reported to be mobile, with an average main-chain temperature factor of 45 Å^2^, compared with mean values of 28 and of 32 Å^2^ for main-chain atoms of the helix domain and for all main-chain atoms, respectively. The subunit interface is primarily made up by the helix and tail domains of the two subunits, with only residues 130–132 being from the coenzyme domain. A network of ordered water molecules at the interface is important in stabilizing the dimer (Phillips *et al.*, 1995 ▸). Also, acetylation of Lys294 (Lys293 in the sheep structure) by acetylCoA acetyltransferase 2 has been demonstrated to upregulate 6PGDH in cancer cells by means of stabilization of the dimer (Shan *et al.*, 2014 ▸). The importance of the tail in modulating the homodimer function has clearly been revealed by the finding that EGFR activates the phosphorylation of Tyr481 by Fyn kinase in human glioma cells, increasing NADP^+^ affinity and enzyme activity (Liu *et al.*, 2019 ▸). Phosphorylation of 6PGDH has also been reported in cyanobacteria in response to heat stress (Zorina *et al.*, 2011 ▸).

### 3.2. Crystallographic structures showing the asymmetric mechanism and evidence of an open and a closed 6PGDH subunit conformation

The second solved crystallographic structure of 6PGDH, that from the pathogenic protist *T. brucei* (PDB entry 1pgj), was also determined in the laboratory of M. J. Adams. This had a dimer in the asymmetric unit (Phillips *et al.*, 1998 ▸). Some differences were seen between the two *T. brucei* subunits, while the overall structure of the subunit is like that of the sheep enzyme, despite only 35% amino-acid sequence identity. The triplet Asn32, Arg33 and Thr34 binding the 2′-phosphate and the adenine ribose is conserved, although here a glycine replaces Ala11 in the fingerprint. Not only is the *T. brucei* 6PGDH specific for NADP^+^, but it also shows a much higher affinity for the coenzyme compared with the mammalian enzyme (Hanau *et al.*, 1996 ▸). The Ala–Gly replacement, as well as that of Lys75 by Gln77, allow more contacts between the enzyme and the bisphosphate, as shown by the 40-fold higher *K*_i_ for 2′5′-ADP of sheep 6PGDH compared with the *T. brucei* enzyme. An additional hydrogen bond to an adenine N atom should also be allowed, with Thr85 replacing Phe83, and this closes the adenine pocket in the sheep enzyme (Phillips *et al.*, 1998 ▸).

While the coenzyme domains come into closer contact in the parasite enzyme, with a 7° rotation compared with sheep 6PGDH, this domain differs in the two subunits, above all in the βD–αD loop and in the conformation of Arg32 (Phillips *et al.*, 1998 ▸). Other crystallographic structures showing asymmetry have subsequently been solved. The structure of *L. lactis* 6PGDH has been solved in complex with both Ru5P and NADP^+^ (PDB entry 2iyp) and in complexes with the high-energy reaction intermediate analogues 4-phospho-d-erythronohydroxamic acid (PEX) or 4-phospho-d-erythron­amide (PEA) and the coenzyme lacking nicotinamide, its bound ribose and α-phosphate (A2P) (PDB entries 2iz0 and 2iz1, respectively). In these crystals three subunits were present in the asymmetric unit, with two subunits forming one noncrystallographic symmetry-related dimer and the remaining monomer as the single monomer seen in the 6PG complex of the *L. lactis* enzyme, which can form a functional dimer using a twofold crystallographic axis of symmetry (PDB entry 2iyo; Sundaramoorthy *et al.*, 2007 ▸). In addition, only one of the three subunits (subunit *A*) contained the ternary complex, which is in agreement with the half-of-the-sites reactivity (Rippa *et al.*, 1979 ▸; Dallocchio *et al.*, 1981 ▸; Hanau *et al.*, 1992*b* ▸).

**Figure 9:**
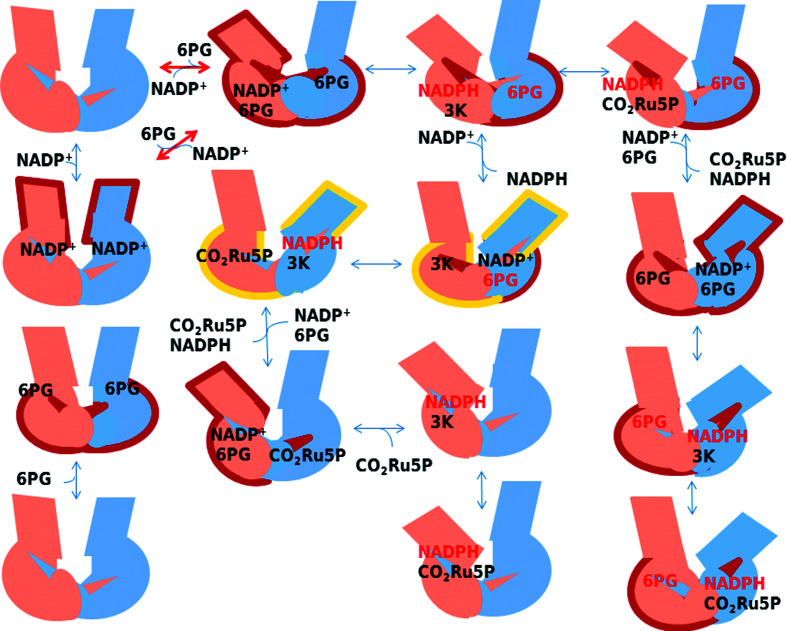
Envisioned figurative model of how 6PGDH works. The two subunits are depicted in different colours. Two possible conformations, symmetrical and asymmetrical, are represented. In the asymmetrical configuration one subunit has the coenzyme-binding domain rotated compared with the other domain, allowing entrance/release of the coenzyme. Red arrows indicate transitions between the symmetrical and asymmetrical 6PGDH conformations. Ligands functioning as decarboxylation and Ru5P-binding activators are shown in red. The two structures outlined in yellow have both subunits involved in catalysis: one in the redox reaction and the other in decarboxylation.

Superpositions of both subunit *A* and the subunit in the 6PG binary complex, relative to subunits *B* and *C*, show a movement (5° rotation) of the coenzyme domain (schematized in Fig. 9 ▸; details of the figure are explained in Section 5) like that seen on superposition of the coenzyme-binding domains of the sheep and *T. brucei* 6PGDH structures. The largest main-chain difference between the sheep and *T. brucei* enzymes is 2.1 Å in one of the NADP^+^-binding domains, while it is 1.6 Å between subunit *A* of the *L. lactis* enzyme and the other subunits (Phillips *et al.*, 1998 ▸; Sundaramoorthy *et al.*, 2007 ▸). However, such magnitudes of shift are rather close to coordinate error levels, if the shift is considered at a 3σ level of significance. We found that the *K*_d_ for both NADPH and NADP^+^ was two orders of magnitude lower in the presence of the intermediate analogue 4PE, decreasing from 7 µ*M* to 40 n*M*, for *T. brucei* 6PGDH (Montin *et al.*, 2007 ▸). Cervellati and coworkers suggested that the conformational changes could be related to the presence of the two (closed and open) forms and be consistent with the requirement of both 6PG and NADP^+^ to generate a closed conformation (Cervellati *et al.*, 2008 ▸). However, both the 6PG activation of the decarboxyl­ation step, suggesting a reciprocating sites mechanism, and the 6PG-induced enzyme reactivity change (see Section 2) indicate that the substrate alone might induce an occluded conformation. However, differences may exist due to a species-specific pattern and/or diverse experimental pH conditions (Rippa *et al.*, 1998 ▸).

**Figure 10:**
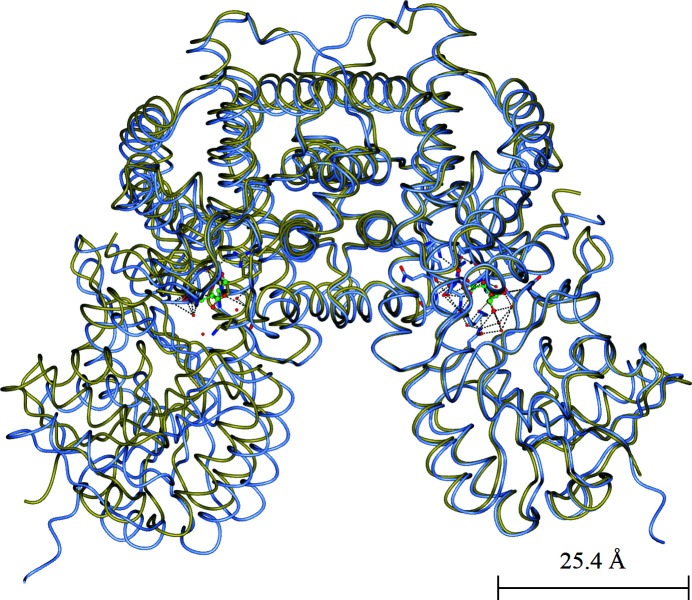
Overlay of the *G. stearothermophilus* 6PGDH–6PG complex (PDB entry 2w90, pale blue) onto the *E. coli* 6PGDH–glucose complex (PDB entry 2zyd, gold) via subunit *A*. An 8 Å shift is shown at the bottom left, with PDB entry 2zyd more open, when the *A* subunits are overlaid at the right. This figure was prepared in *CCP*4*mg* (McNicholas *et al.*, 2011 ▸).

**Figure 11:**
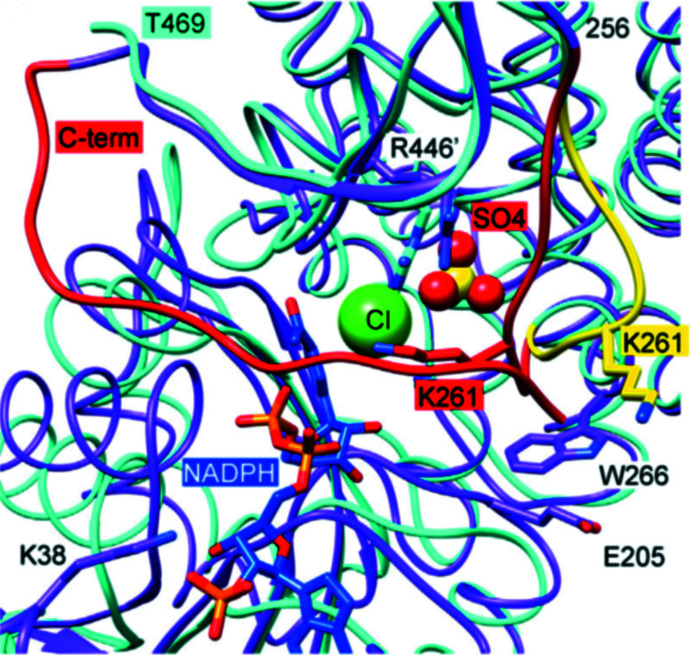
Active-site loop of human 6PGDH in the open (yellow) and closed (red) conformations from superimposition of the apoenzyme (light blue; PDB entry 4gwg) and the NADPH complex (magenta; PDB entry 2jkv), where the substrate-binding site is occupied by a sulfate and a chloride ion [reproduced from Haeussler *et al.* (2018 ▸) with permission from Elsevier].

**Figure 12:**
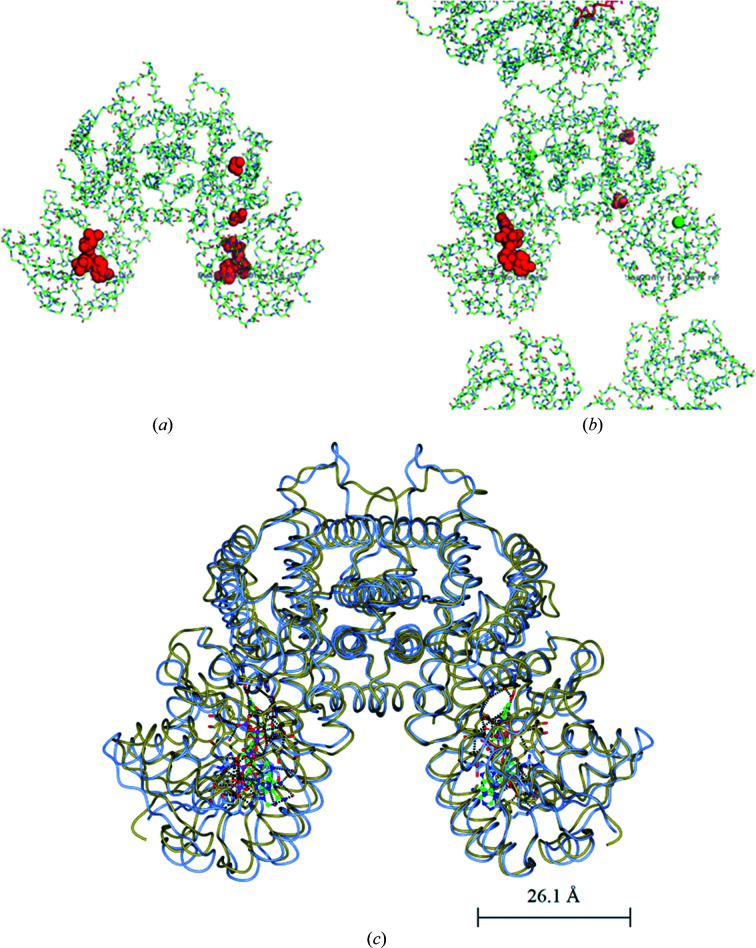
(*a*) *P. falciparum* NADP^+^–6PGDH complex (PDB entry 6fqy). (*b*) Human NADPH–6PGDH complex (PDB entry 2jkv). Ligands are shown in sphere mode in (*a*) (two NADP^+^ and two ethanediol molecules) and in only one of the homodimers in (*b*) (one NADPH molecule, two sulfate ions and two chloride ions in green). (*c*) Overlay of one homodimer from PDB entry 2jkv (gold) with NADPH bound in both subunits onto that from PDB entry 6fqy (pale blue), showing a 3.5 Å opening of the coenzyme domain in the left subunit of PDB entry 2jkv when both subunits are overlaid at the right. (*c*) was prepared in *CCP*4*mg* (McNicholas *et al.*, 2011 ▸).

When the *E. coli* 6PGDH–glucose complex (PDB entry 2zyd) is superposed on the 6PGDH–6PG complex (PDB entry 2zya) to assess the relative movements in each dimer, a 0.8 Å rotational shift of one subunit of the first complex relative to the other complex appears when the other two subunits are overlaid using the LSQ function in *Coot* (Emsley *et al.*, 2010 ▸). In contrast, the same LSQ overlay calculation for the *Geobacillus stearothermophilus* 6PGDH–6PG complex (PDB entry 2w90) and the *E. coli* 6PGDH–glucose complex (PDB entry 2zyd) shows an 8 Å shift of the N-terminal domain of one subunit about the join of the two monomers at the all-helix domains, with PDB entry 2zyd being the more open structure (Fig. 10 ▸). The crystallographic structure of *P. falciparum* 6PGDH, while not showing part of the NADP^+^ in the NADP^+^–6PGDH complex, implying flexibility of the ribose and the nicotinamide moiety, presents the cofactor-binding domain of one subunit rotated by 5° compared with the other (PDB entry 6fqy) and those in the complex with 6PG (PDB entry 6fqz) or the apoenzyme (PDB entry 6fqx), even if the largest root-mean-square deviation (r.m.s.d.) between the NADP^+^ complex and the other structures is 1 Å for 936 C^α^ atoms. In addition, the main finding is that a flexible loop close to the active site comprising residues 255–262 adopts a closed conformation upon the binding of 6PG, with differences between the C^α^ positions of up to 3.7 Å (PDB entry 6fqz; Haeussler *et al.*, 2018 ▸). Haeussler and coworkers compared the published structures of 6PGDH, reporting that the open loop conformation is only visible in their apoenzyme (PDB entry 6fqx) and NADP^+^ complex, in the *Klebsiella pneumoniae* (PDB entry 2zyg) and human (PDB entry 4gwg) apoenzymes and in the human 3-phosphoglycerate (3PG) complex (PDB entry 4gwk), in which the inhibitor forces the active-site loop into the open form (Fig. 11 ▸). The closed form is adopted even if only an anion interacts with the positively charged Arg446 (numbering as in the sheep sequence) and with loop residues in the substrate-binding site (Fig. 11 ▸). In the substrate-binding site of the human NADPH complex (PDB entry 2jkv) a sulfate and a chloride ion are present; therefore, the loop adopts an occluded conformation (Fig. 11 ▸) and at the same time the long C-terminus, which is disordered in the open form, reorganizes to cover the active site (Haeussler *et al.*, 2018 ▸). Based on crystallographic data and structural comparison, they suggest that in *P. falciparum* 6PGDH only 6PG binds in the catalytically relevant position and that the conserved Trp265 and the parasite-specific Trp104 are important in linking the NADP^+^ and 6PG domains, allowing the loop to lock, which protects the active site from solvent. The importance of the main-chain amide of Lys261 (Lys260 and Lys262 in the sheep and *L. lactis* structures, respectively) in binding the 6-phosphate was indicated in the sheep 6PGDH structure, and subsequently it was pointed out that once the phosphate has bound in the *L. lactis* crystal structure, Lys262 covers the active site (Adams *et al.*, 1994 ▸; Sundaramoorthy *et al.*, 2007 ▸). In Fig. 12 ▸ two 6PGDH structures are shown; the human 6PGDH–NADPH complex (PDB entry 2jkv), in which a NADPH is bound in only one subunit in one dimer (Fig. 12 ▸ *b*, two sulfates and two chloride ions are also shown), and the *P. falciparum* 6PGDH–NADP^+^ complex (PDB entry 6fqy), with the coenzyme bound in each of the two subunits of the dimer and two ethanediol molecules in only one of the two subunits (Fig. 12 ▸ *a*). The superposition of PDB entry 2jkv onto PDB entry 6fqy in *CCP*4*mg* (McNicholas *et al.*, 2011 ▸) demonstrates a 3.5 Å opening of the coenzyme domain in only one of the two NADPH-bound subunits of the human enzyme relative to the superimposed subunit of the *P. falciparum* NADP^+^-bound 6PGDH (Fig. 12 ▸ *c*).

Also, the structure of *E. coli* 6PGDH in complex with 6PG or glucose or with both 6PG and ATR (coenzyme devoid of nicotinamide and bound ribose) (PDB entries 2zya, 2zyd and 3fwn, respectively) had a dimer in the asymmetric unit (Chen *et al.*, 2010 ▸). In the complex with 6PG and ATR, two 6PG molecules were bound per dimer, but only one ATR, in agreement with the half-sites reactivity of coenzyme analogues that is found during ternary-complex formation in the yeast, sheep and *T. brucei* 6PGDHs (Rippa *et al.*, 2000 ▸; Montin *et al.*, 2007 ▸). Chen and coworkers reported a coenzyme domain rotation of 10.4° in the subunit with the ternary complex, corresponding to an opening movement of 7.3 Å (Chen *et al.*, 2010 ▸). The crystal structures of sheep 6PGDH showed sulfates displaced by 6PG, and various polyanions have been shown to be competitive inhibitors of 6PGDH, including sulfate, phosphate, pyrophosphate, citrate, tetravanadate, trinitrobenzenesulfonate and similar compounds, and phosphonates (Adams *et al.*, 1994 ▸; Phillips *et al.*, 1995 ▸; Bergamini *et al.*, 1995 ▸; Hanau *et al.*, 1993*b* ▸, 1996 ▸, 2007 ▸). This is suggested to be the reason why two possible conformations of the enzyme were seen when crystallization was not performed in sulfate or similar polyanionic solvents. These configurations may well resemble those seen in complexes with physiologically relevant ligands.

All of the solved ternary complexes confirmed the conserved lysine and glutamate to be the residues involved in the acid–base mechanism of 6PGDH. They also confirmed the presence of the Ser–His–Asn triad (Ser128, His186 and Asn187 in the sheep 6PGDH sequence) linking the coenzyme- and 6PG-binding sites. Mutagenesis showed the importance of these residues in both 6PG and NADPH binding, and in all steps of catalysis, including the precatalytic isomerization (Li *et al.*, 2006 ▸).

## 4. Functional oligomerization

### 4.1. Exchange of the C-terminus between subunits

Maturana *et al.* (2021 ▸) showed that the structural difference between 6PGDH and HIBADH is the exchange of the C-terminal α-helix between subunits. For this exchange, the short-chain 6PGDH needs to be tetrameric. In fact, the only short-chain 6PGDH which is dimeric is the membrane-associated *Gluconacetobacter diazotrophicus* 6PGDH. Here, the substrate-binding site is at the interface and is made up of residues from both subunits, but without tail swapping, as in the HIBADHs (Lokanath *et al.*, 2005 ▸; Park *et al.*, 2016 ▸; Sarmiento-Pavía *et al.*, 2021 ▸; Maturana *et al.*, 2021 ▸). This acetic acid bacterium possesses an Asp–Arg–Asp motif in the β2–α2 loop in place of the Asn–Arg–Thr motif, thus preferring NAD^+^ over NADP^+^. Sarmiento-Pavía *et al.* (2021 ▸) propose that the produced NADH diffuses to the periplasmic oxidase via an NADH respiratory-chain dehydrogenase, thus sensing inner catabolism, since a particular PPP is the main pathway in these bacteria. In all other 6PGDHs residues of the tail participate in the formation of the substrate-binding site, such as the conserved Arg446 and His452 (sheep numbering).

### 4.2. Two groups in the long-chain 6PGDH family

The tail is the domain which shows the largest differences between different species. It consists either of a single helix or of a helix and a loop, while in the *G. stearothermophilus* enzyme the helix is followed by two β-strands (Fig. 6 ▸; Adams *et al.*, 1994 ▸; Cameron *et al.*, 2009 ▸; Sarmiento-Pavía *et al.*, 2021 ▸). In the long-chain 6PGDHs, two groups can be recognized: those with a glycine/serine-rich C-terminus that is about 15 residues longer and those without. The second group typically has a charged residue very close to the C-terminus that forms a salt bridge to a residue in αf (Phillips *et al.*, 1998 ▸). The human, sheep and *S. cerevisiae* 6PGDHs belong to the first group, while those from many microrganisms belong to the second group. Although interactions between the hydrophobic groups of the central helix domain of each monomer play a major role in dimerization, some of the ionized residues of the highly charged tail of 6PGDH from *T. brucei* and similar protists form inter-subunit salt bridges that contribute to protein stability and a larger monomer–monomer contact area compared with the other 6PGDHs (around 6200 Å^2^ in the *T. brucei* enzyme; Phillips *et al.*, 1998 ▸; Igoillo Esteve & Cazzulo, 2004 ▸; He *et al.*, 2007 ▸; González *et al.*, 2011 ▸).

### 4.3. Oligomerization equilibrium

Using various techniques applied to the enzyme in solution, a dimer–tetramer equilibrium was found for *T. brucei* 6PGDH, with a specific activity of the tetramer that was more than three times higher than that of the dimer. Ligands strongly affect the oligomerization kinetics, with NADPH promoting the tetramer, while NADP^+^ and 6PG cause a shift towards the dimer (Hanau *et al.*, 2013 ▸). This again could suggest the presence of at least two conformations, with the conformation binding NADPH being more prone to tetramerization. Several mutants of the catalytic residues (Glu192Gln, Lys185His and His188Leu) are even more prone to tetramerization (unpublished data). On the other hand, the fact that the sheep enzyme does not show this dimer–tetramer equilibrium under the same conditions agrees with the phylogenetic tree of 6PGDH sequences reported by Sarmiento-Pavía *et al.* (2021 ▸), in which *T. brucei* 6PGDH is on the border between the two well differentiated clusters of small-chain (tetrameric) and long-chain (dimeric) 6PGDHs. Some of the other peculiarities of this parasite 6PGDH have already been cited in Section 3.2. While it is a long-chain 6PGDH and has the Asn-Arg-*X* motif typical of NADP^+^-specific 6PGDHs (the second cluster in the phylogenetic tree), it also presents the typical NAD^+^-specific Gly-*X*-Gly-*X*-*X*-Gly fingerprint found in the mainly tetrameric first cluster (Phillips *et al.*, 1998 ▸; Sarmiento-Pavía *et al.*, 2021 ▸). Moreover, other differences between the parasite and mammalian 6PGDH enzymes are the seven β-strands in the coenzyme domain in place of the eight found in most 6PGDHs, and two small 3_10_-helices in the central domain that are not present in the mammalian 6PGDH. It is recognized that the *T. brucei* 6PGDH sequence shows more similarity to those of plant and cyanobacterial 6PGDHs than the mammalian enzymes. It is not clear whether this is due to lateral gene transfer or primary endosymbiotic gene transfer early in evolution followed by the loss of either the pre-existing or cyanobacterial gene (Krepinsky *et al.*, 2001 ▸; Maruyama *et al.*, 2008 ▸; Maturana *et al.*, 2021 ▸).

Nonetheless, lysine acetylation in EGF-stimulated cells and human cancer has been shown to affect oligomerization (dimerization) and to upregulate human 6PGDH (Shan *et al.*, 2014 ▸). Cytosolic NADP^+^-binding malic enzyme (ME1) is a homotetramer in which the subunit structure adopts a similar conformation to that of the 6PGDH subunit, with a long tail containing one helix that protrudes away and inserts into two other subunits, as seen in the crystal structures (Hsieh *et al.*, 2014 ▸). It has also been shown that ME1 can form hetero-oligomers with 6PGDH that enhance its activity in cancer or immortalized cells (Yao *et al.*, 2017 ▸). Thus, an equilibrium between an inactive monomer and an active oligomer also exists for human 6PGDH. Malic enzyme (ME) and 6PGDH, and also isocitrate dehydrogenase (IDH), catalyse oxidative decarboxylations and a final tautomerization step. They also have half-of-the-sites reactivity and similar kinetics. The homo-oligomeric structures of all three enzymes present subunits with C-terminal domain interlocking, although a significant difference between 6PGDH and the others is that ME and IDH require a bivalent cation for activity (Rippa *et al.*, 2000 ▸; Chang & Tong, 2003 ▸; Xu *et al.*, 2004 ▸). Thus, 6PGDH can form a supramolecular complex with G6PDH, increasing the efficiency of NADPH production, as shown in human neutrophils (Kindzelskii *et al.*, 2004 ▸). To provide the necessary NADPH for proliferation and survival, cancer cells are able to form active hetero-oligomers with ME1, the subunit of which in some ways mimics that of 6PGDH (Yao *et al.*, 2017 ▸). For IDH and ME it has been shown that more conformations exist, including an open and a closed conformation, that correspond to distinct functional states and bound ligands (Gonçalves *et al.*, 2012 ▸; Hsieh *et al.*, 2014 ▸). In both cases, the induced fit needed for catalysis consists of a hinge motion allowing new residues to interact, closing the active site.

## 5. A model for 6PGDH function

By way of a comparison of the solved 6PGDH crystal structures discussed in this topical review, and of the known enzyme properties (based on the many references reported here), a schematic model for 6PGDH is envisioned as follows (Fig. 9 ▸), in which

(i) the enzyme can have both a symmetrical and an asymmetrical configuration (red arrows indicate transitions between the two 6PGDH conformations),
(ii) in the asymmetrical configuration one subunit of the homodimeric 6PGDH could have the coenzyme-binding domain rotated compared with the other, allowing entrance/release of the coenzyme,
(iii) 6PGDH is asymmetrical when a ternary complex is present,
(iv) 6PGDH can also be asymmetrical with NADPH bound,
(v) only half of the subunits bind the coenzyme during ternary-complex formation,
(vi) 6PG and NADPH cannot bind simultaneously to the same subunit,
(vii) subunits are not involved in the same catalytic step at the same time,
(viii) either 6PG or NADPH can activate the decarboxyl­ation step using different mechanisms (shown in red in the Fig. 9 ▸),
(ix) when NADPH is immediately released as the first product, 6PG is the decarboxylation activator and both subunits in the dimer can be involved in catalysis, one in the redox reaction and the other in decarboxylation (enzyme outlined in yellow in Fig. 9 ▸), allowing the enzyme to work at the full rate (Hanau *et al.*, 1993*a* ▸, 1996 ▸; Rippa *et al.*, 2000 ▸),
(x) it is conceivable that the asymmetrical configuration is the full-rate catalytic configuration and
(xi) it is also conceivable that the single subunit can have at least two different configurations, including a closed and an open configuration.

Not all possible dimer structures are represented in Fig. 9 ▸; those that are missing have the same shape and bound ligands but would have the inverted subunit colours. Moreover, Fig. 9 ▸ is a concise and simplified model in which the two chemical steps of decarboxylation and enol–keto tautomerization are not distinguished but are represented as only one stage.

It is not possible to state whether the asymmetric configuration is the so-called ‘open’ or ‘closed’ configuration, and anyway this would be an absolute simplification. As the *P. falciparum* crystal studies showed, active-site loop flexibility is also involved in reaching the catalytic configuration (Fig. 11 ▸).

From the superpositions shown in this review of the entire homodimeric enzyme structures, two 6PGDH crystal structures are shown to be slightly more open. The *E. coli* 6PGDH–glucose complex is more open relative to the 6PG complex, as most apparent for the *G. stearothermophilus* complex (Fig. 10 ▸), and the human NADPH complex is more open relative to *P. falciparum* 6PGDH bound to a large part of NADP^+^ (Fig. 12 ▸). In other terms, the analysed 6PGDH–6PG and 6PGDH–NADP^+^ complexes result in more closed crystal structures.

## 6. Conclusions

To summarize, many crystal structures of 6PGDH are now present in the PDB. These are from several microorganisms through to the human enzyme. Nevertheless, yet more structural and computational modelling studies will surely be useful to add insight into the mechanics of the coexisting catalytic and cooperative mechanisms of this interesting enzyme. There have been diverse studies complementary to the crystal structures: mutagenesis, the use of inhibitors including polyfunctional and covalent modifiers, kinetic and binding studies, post-translational modification and research on the enzyme in cancer cells, as well as bio­nanotechnological applications. From the first crystal structure it was evident that communication between subunits is allowed at the active site since it is made up of residues from both subunits. One of the most recently solved 6PGDH structures, that from *P. falciparum*, confirms that Lys260 and Arg446, which were shown to bind the substrate 6-phosphate in the sheep 6PGDH complex, are crucial. Thus, the tail is involved in the induced fit of 6PG, although the subsequent precise chemical steps and inter­actions are not yet understood.

Developments in cryogenic electron microscopy (cryoEM) are interesting, with lower molecular weights now being reached. Therefore, this method might reveal further enzyme intermediate states without ‘forcing’ the protein into a crystal. The unusual shape (like a butterfly) and the dimer molecular weight (100 kDa) of the enzyme might be within reach of cryoEM, thereby further revealing the flexibility of the enzyme, *i.e.* its dynamic intermediate states. Also, neutron protein crystallography would permit determination of the protonation states of the enzyme. Small-angle X-ray scattering (SAXS) would give results on the enzyme in solution at room temperature, *i.e.* at a more physiologically relevant temperature, and would greatly help in elucidating the mechanism and in assays to determine whether the described model (Fig. 9 ▸) applies.

A curious finding is that 6PGDH is not only homo-oligomeric but also hetero-oligomeric under specific conditions (Yao *et al.*, 2017 ▸). We hope that as a consequence of this review further interest will arise in 6PGDH, not least as it can be considered as a therapeutical target in immunity, cancer and infectious diseases as well as having potential in bio­nanotechnology, in addition to its fundamental scientific interest in structural chemistry, biology and biochemistry.

## Figures and Tables

**Table 1:** Crystallographic structures cited in this article NR, not reported; AU, asymmetric unit.

Name; PDB code; organism; references	Crystallization details	Resolution (Å); space group; No. of protein molecules in AU†	The highest ± difference Fourier electron-density peak and any specific comments‡	PDB validation assessment (clashscore; specific comments based on the PDB report)
6PGDH; 2pgd; *O. aries*; Phillips *et al.* (1995 ▸)	Ammonium sulfate, 50 m*M* potassium phosphate, 1 m*M* EDTA, 288.0 K, pH 6.5	2.00; *C*222_1_; 1	8.5σ. There are 27 peaks above ±5σ, the *Coot* default threshold. These are nearly all minor adjustments needed to side chains or split-occupancy waters.	3
6PGDH–6PG; 1pgp; *O. aries*; Adams *et al.* (1994 ▸)	As above	2.50; *C*222_1_; 1	5.7σ; 6 peaks above ±5σ; 3 new bound waters, 2 minor side-chain adjustments	3
6PGDH–NADPH; 1pgo; *O. aries*; Adams *et al.* (1994 ▸)	As above	2.50; *C*222_1_; 1	7.6σ; 5 peaks above ±5σ. Top 2 peaks suggest a possible adjustment to the nicotinamide ring.	8. These are predominantly H-atom clashes.
6PGDH–Nbr^8^ADP; 1pgn; *O. aries*; Adams *et al.* (1994 ▸)	As above	2.30; *C*222_1_; 1	−5.3σ; 3 peaks above ±5σ.	8. These are predominantly H-atom clashes.
6PGDH–2′AMP; 1pgq; *O. aries*; Adams *et al.* (1994 ▸)	As above	3.17; *C*222_1_; 1	−6.4σ; 2 peaks above ±5σ.	8. These are predominantly H-atom clashes. 〈*I*/σ(*I*)〉 = 5 at the resolution edge (3.15 Å); unsure why data were truncated at this resolution limit.
6PGDH; 1pgj; *T. brucei*; Phillips *et al.* (1998 ▸)	Ammonium sulfate, 50 m*M* potassium phosphate, 5 m*M* DTT, 293.0 K, pH 7.0	2.82; *P*3_1_21; 2	5.3σ; 3 peaks above −5σ.	14. These are predominantly H-atom clashes.
HIBADH; 2cvz; *T. thermophilus*; Lokanath *et al.* (2005 ▸)	PEG 4K, 0.1 *M* Tris–HCl, 1 *M* lithium chlorate, 295.0 K, pH 8.10	1.80; *P*2_1_2_1_2_1_; 4	18.8σ. 164 peaks above ±5σ. The top 3 peaks are all negative, of similar magnitude and on the MSE seleniums in subunits *A*, *B* and *C*. Other negative peaks are also on Se atoms. Evidence of irradiation damage to Asp and Glu side chains. Other peaks probably bound waters to be assigned and side chains needing adjustment.	6. 〈*I*/σ(*I*)〉 = 4.33 at the resolution edge (1.8 Å); unsure why data were truncated at this resolution limit.
6PGDH–Ru5P–NADP^+^; 2iyp; *L. lactis*; Sundaramoorthy *et al.* (2007 ▸)	0.1 *M* sodium cacodylate, 300 m*M* ammonium acetate, 25%(*w*/*v*) PEG 3350, 100.0 K, pH 7.2	2.79; *C*121; 3	7.5σ; 18 peaks above −5σ. These are likely to be further bound waters and a few possible solute molecules.	8. These are predominantly H-atom clashes.
6PGDH–PEX/PEA–A2P; 2iz0; *L. lactis*; Sundaramoorthy *et al.* (2007 ▸)	As above	2.60; *C*121; 3	9.8σ. 48 peaks above ±5σ. These are likely to be split-occupancy waters, some difficult to interpret solute molecules and WatA2128 with a *B* factor of 2 Å^2^. There are 28 waters with *B* factors of 2 Å^2^ which are very likely to be incorrectly assigned.	3. 〈*I*/σ(*I*)〉 is 4.6 at the resolution limit of 2.6 Å; unsure why data were truncated at this resolution limit.
6PGDH–PEX/PEA–A2P; 2iz1; *L. lactis*; Sundaramoorthy *et al.* (2007 ▸)	As above	2.3; *C*121; 3	9.35σ. 81 peaks above ±5σ. Quite a number of Glu side chains showing irradiation damage. Also more possible bound waters or side-chain adjustments.	5. 〈*I*/σ(*I*)〉 = 8.85 at the resolution limit of 2.29 Å; unsure why data were truncated at this resolution limit.
6PGDH–6PG; 2iyo; *L. lactis*; Sundaramoorthy *et al.* (2007 ▸)	As above	2.4; *P*3_2_12; 1	7.6σ. 26 peaks above ±5σ. Quite a number of Asp and Glu side chains showing irradiation damage. Also more possible bound waters or side-chain adjustments.	4. 〈*I*/σ(*I*)〉 = 5.5 at the resolution limit of 2.4 Å; unsure why data were truncated at this resolution limit.
Gnd1; 2p4q; *S. cerevisiae*; He *et al.* (2007 ▸)	1.28 *M* sodium citrate, 288.0 K, pH 6.5	2.37; *P*6_5_22; 1	8.7σ. 32 peaks above ±5σ. Various Ile side-chains need repositioning. Some unfitted solute molecules and split-occupancy side chains.	17. Mainly H-atom clashes. Ile side-chain repositioning may reduce clashscore.
6PGDH–6PG; 2w90; *G. stearo­thermophilus*; Cameron *et al.* (2009 ▸)	0.2 *M* lithium sulfate, 2.2 *M* ammonium sulfate, temperature NR, pH 7.4	2.20; *P*2_1_2_1_2_1_; 2	−9.0σ. 34 peaks above ±5σ, of which 32 are negative; may be due to irradiation damage.	7
6PGDH–NADPH; 2jkv; *H. sapiens*; Ng *et al.* (unpublished work)	0.2 *M* sodium sulfate, 20% PEG 3350; 10% *N*-ethylglycine, temperature and pH NR	2.53; *P*12_1_1; 6	9.6σ. 89 peaks above ±5σ. Above 6σ (41 peaks) these are mainly unfitted solute molecules and waters, but some side-chain adjustments are also needed.	6
6PGDH; 4gwg; *H. sapiens*; Hitosugi *et al.* (2012 ▸)	14% PEG 3350, 289.0 K, pH 6.0	1.39; *P*4_3_2_1_2; 1	9.6σ. 70 peaks above ±5σ. 28 peaks checked above 6σ which are bound waters, solute molecules and side-chain adjustments that are needed.	3
6PGDH–3PG; 4gwk; *H. sapiens*; Hitosugi *et al.* (2012 ▸)	As above	1.53; *P*4_3_2_1_2; 1	7.6σ. 55 peaks above ±5σ. 21 peaks checked above 6σ are solute molecules, disorder for residues 308 and 309 and bound waters.	3
6PGDH; 2zyg; *K. pneumoniae*; Chen *et al.* (2010 ▸)	0.12 *M* diammonium hydrogen citrate, 20% PEG 3350, 277.0 K, pH 5.0	2.10; *P*3_2_21; 2	6.7σ. 15 peaks above ±5σ.	9. These are predominantly H-atom clashes.
6PGDH–6PG; 2zya; *E. coli*; Chen *et al.* (2010 ▸)	0.1 *M* trisodium citrate, 0.5 *M* ammonium acetate, 6–7% PEG 3350, 17–18% PEG 4000, 291.0 K, pH 5.4	1.6; *P*2_1_2_1_2_1_; 2	12.1σ. 125 peaks above ±5σ. The 40 peaks above 6σ were checked and are dominated by minor adjustments needed to side chains and also some irradiation damage to side chains and solute molecules. Possible adjustment of 2 6PG molecules.	7. This clashscore would likely improve if the difference-map details of the side chains were attended to.
6PGDH–6PG–ATR; 3fwn; *E. coli*; Chen *et al.* (2010 ▸)	As above	1.5; *P*2_1_2_1_2_1_; 2	−12.1σ. 66 peaks above ±5σ. Top 4 peaks (2 negative, 2 positive) suggest the phosphate of 6PG subunit *B* could be adjusted. The peaks above 6σ are mainly side chains that could be modelled as split occupancy or minor adjustments made and bound waters.	10. The top six clashes involve water O atoms albeit with side-chain H atoms.
6PGDH–glucose; 2zyd; *E. coli*; Chen *et al.* (2010 ▸)	As above	1.5; *P*2_1_2_1_2_1_; 2	9.8σ. 85 peaks above ±5σ. Above 6σ the 26 peaks mainly show the need for split-occupancy side chains; there is also some main-chain disorder but this is probably hard to model.	10. Water O atoms and glycerol A3929 could be revisited to improve the clashscore.
Pf6PGD; 6fqx; *P. falciparum*; Haeussler *et al.* (2018 ▸)	25% PEG 4000, 15% glycerol, 0.085 *M* sodium citrate, 0.17 *M* ammonium acetate, 295.0 K, pH 5.6	2.80; *P*6_5_; 8	−8.5σ. 69 peaks above ±5σ. Simple repositioning of the Phe372E side chains would deal with the top 2 peaks. Several side-chain adjustments and solute molecules are needed.	12. 〈*I*/σ(*I*)〉 = 0.75 at 2.8σ and 8 subunits in the AU increase the clashscore to the higher value of 12. The Laue group was checked using *POINTLESS* (Evans, 2011 ▸) and confirmed as 6/*m* (*i.e.* not 6/*mmm*).
Pf6PGD–NADP^+^; 6fqy; Haeussler *et al.* (2018 ▸)	25% PEG MME 550, 0.1 *M* HEPES, 295.0 K, pH 4.6	2.90; *P*3_2_21; 2	6.2σ. 5 peaks above ±5σ. The 2 NAPs (A501 and B500) are truncated and do not include their nicotinamide ring or ribose (there is also no electron density for them).	14. 〈*I*/σ(*I*)〉 = 0.83 at 2.9 Å. The Laue group and screw axis were checked using *POINTLESS* and confirmed.
Pf6PGD–6PG; 6fqz; *P. falciparum*; Haeussler *et al.* (2018 ▸)	24% PEG 1500, 20% glycerol, 295.0 K, pH NR	1.90; *P*3_2_21; 2	−8.0σ. 23 peaks above ±5σ, but only 2 above 6σ.	3. 〈*I*/σ(*I*)〉 = 0.5 at 1.9 Å resolution.
Silver-bound 6PGDH; 7cb6; *S. aureus*; Wang *et al.* (2021 ▸)	0.1 *M* sodium nitrate, 0.2 *M* ammonium nitrate, 0.1 *M* MES–Na, 50% PEG 3350, 298.0 K, pH 6.5	2.64; *P*2_1_2_1_2_1_; 4	8.5σ. 76 peaks above ±5σ. Difference-map activity at several Cys residues which may be due to binding of the Ag atoms and the disorder associated with these.	4. The Laue group and screw axes were checked using *POINTLESS* and confirmed.

† For the entries up to PDB entry 2zyd with more than one subunit in the AU, the space groups were confirmed using *Zanuda* (Lebedev & Isupov, 2014 ▸).

‡ The validation report from the PDB concerns the derived model and not unmodelled peaks. The *F*_o_ − *F*_c_ map was inspected in the *Coot* visualization system (Emsley *et al.*, 2010 ▸) to describe the unmodelled peaks.
